# Relationships between mechanical properties and drug release from electrospun fibers of PCL and PLGA blends

**DOI:** 10.1016/j.jmbbm.2016.09.004

**Published:** 2017-01

**Authors:** Shih-Feng Chou, Kim A. Woodrow

**Affiliations:** Department of Bioengineering, University of Washington, 3720 15th Ave NE, Seattle, WA 98195-5061, USA

**Keywords:** Electrospun fibers, Mechanical properties, Drug loading, Drug release, Drug–polymer interaction, Drug partition

## Abstract

Electrospun nanofibers have the potential to achieve high drug loading and the ability to sustain drug release. Mechanical properties of the drug-incorporated fibers suggest the importance of drug–polymer interactions. In this study, we investigated the mechanical properties of electrospun polycaprolactone (PCL) and poly (D,L-lactic-*co*-glycolic) acid (PLGA) fibers at various blend ratios in the presence and absence of a small molecule hydrophilic drug, tenofovir (TFV). Young׳s modulus of the blend fibers showed dependence on PLGA content and the addition of the drug. At a PCL/PLGA (20/80) composition, Young׳s modulus and tensile strength were independent of drug loading up to 40 wt% due to offsetting effects from drug–polymer interactions. In vitro drug release studies suggested that release of TFV significantly decreased fiber mechanical properties. In addition, mechanically stretched fibers displayed a faster release rate as compared to the non-stretched fibers. Finally, drug partition in the blend fibers was estimated using a mechanical model and then experimentally confirmed with a composite of individually stacked fiber meshes. This work provides scientific understanding on the dependence of drug release and drug loading on the mechanical properties of drug-eluting fibers.

## Highlights

• Drug–polymer interactions in electrospun drug-eluting fibers.
• Correlations of drug release, polymer degradation on fiber tensile properties.
• Drug partitioning in blend polyester fibers.

## Graphical abstract

**Figure:**
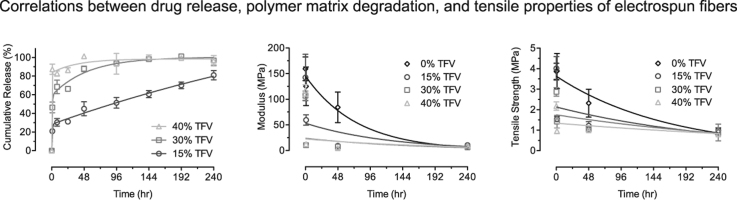

## 1 Introduction

Electrospinning is a process that utilizes an electric field to continuously draw fibers from a viscous polymeric solution through rapid solvent evaporation. One of the advantages in electrospinning is that virtually any polymer can be electrospun into fibers (Huang et al., 2003). The resulting nonwoven fibrous structure along with the versatility of polymer selections and polymer blend combinations makes electrospun fibers an ideal platform for many biomedical applications (Jiang et al., 2015). For example, fibers electrospun from polymer blends of polycaprolactone and polyglyconate were used in tissue engineering as biomaterials to support cell growth where fiber degradation and mechanical properties were dependent on polymer compositions (Schindler et al., 2013). Another promising biomedical application for electrospun fibers of polymer blends is the ability to modulate drug release (Chou et al., 2015). For example, release rate of teriflunomide (up to 15 wt%) from blend fibers of poly(lactic acid) and poly(butylene adipate) was modulated by blend polymer compositions (Siafaka et al., 2016). Furthermore, poly(lactide-co-glycolide), poly(ethylene glycol)-b-poly(lactide), and poly(lactide) (80/5/15) blend fibers showed sustained release of cefoxitin sodium for 7 days as compared to burst release from poly(lactide-co-glycolide) fibers in 6 h (Kim et al., 2004). More importantly, the sustained release behavior of cefoxitin sodium suggested drug entrapment within the hydrophilic block of poly(ethylene glycol)-b-poly(lactide) forming drug/poly(ethylene glycol)-b-poly(lactide) complex, which was further encapsulated in the polymer fibers after electrospinning. These examples demonstrate that fibers comprised of polymer blends have a great potential for tuning drug miscibility and the resulting drug–polymer interactions could lead to different release profiles. While many studies report on drug release from blend fibers, the effect of specific drug–polymer interactions that contribute to the observed release profile has not been investigated. This is perhaps due to the difficulty of measuring drug partitioning in a blended polymer system. In this work, we used mechanical testing as a probe for drug–polymer interactions that could inform drug partitioning in blend fibers of polyesters loaded with a hydrophilic small molecule drug.

The design and function of pharmaceutical electrospun materials may be limited by their relatively low stiffness and strength due to the porous structure. Several factors appear to affect the mechanical properties of electrospun fibers, such as fiber diameters and defects along the fiber axial direction (Chen et al., 2009b; Huang et al., 2004). In addition, fiber alignment has been reported to significantly affect their mechanical properties (Chen et al., 2009a; Kancheva et al., 2014; Lee and Deng, 2012; Nerurkar et al., 2007; Stoyanova et al., 2014). The alignment of fibers provides an alternative strategy in the design of pharmaceutical electrospun materials by tuning the mechanical properties independent of their drug release behaviors. More importantly, mechanical properties may be used to proxy effects of electrospun materials on drug loading, drug–polymer interaction, release behavior, drug partitioning, and polymer degradation. This was demonstrated by incorporation of small molecule drugs and other excipients in fibers that resulted in lowering the crystallinity of semi-crystalline polyester and polyether materials (Yakub et al., 2014). Others have shown that increasing PLGA composition in PCL/PLGA blends decreased the average PCL crystallinity, suggesting the two polymers were miscible and PLGA chains may be disrupting PCL crystalline domains (You et al., 2006). These changes in crystallinity due to drug loading or blend polymers are an indication of drug–polymer interactions and can be directly measured through the mechanical properties of the fibers. For example, tensile strength of poly(lactic-*co*-glycolic acid) fibers significantly decreased from 5% to 10% when increasing bupivacaine HCl loading from 5% to 22% (Weldon et al., 2012). Furthermore, mechanical properties of the poly(lactic-*co*-glycolic acid) fibers decreased due to in vitro polymer degradation, which further increased the release rate of bupivacaine HCl. Therefore, we utilize mechanical testing to infer the effects of drug loading and drug release rates from blend fibers of PCL/PLGA at various compositions.

Here, we study the mechanical performance associated with loading of tenofovir (TFV), a hydrophilic small molecule drug, into fibers of polycaprolactone (PCL) and poly (D,L-lactic-*co*-glycolic) acid (PLGA). We hypothesized that hydrophilic nature of TFV facilitates the loss of mechanical properties during in vitro drug release. PCL/PLGA polymer blends appear to be an ideal system for investigating drug–polymer interactions since our previous study suggested a high compatibility of TFV with solid dispersions of semi-crystalline PCL, amorphous PLGA, and their blends (Carson et al., 2016). Here, we show that increasing drug loading increases drug release rates, and consequently results in a significant loss of mechanical properties. In addition, in vitro release studies from mechanically stretched fibers reveal a much faster release rate than the non-stretched fibers. Drug partitioning in PCL/PLGA blend fibers is further confirmed by experimentally stretching a stacked fiber composition. Our studies contribute significantly to the knowledge of electrospun drug-eluting fibers by correlating the mechanical performance related to drug release and provide important understanding to evaluating these correlations for medical fibers.

## 2 Materials and methods

### 2.1 Materials

Polycaprolactone (PCL) with an average molecular weight (*M*_*w*_) of 80,000 Da was purchased from Sigma-Aldrich (St. Louis, MO, USA). Poly (D,L-lactic-*co*-glycolic) acid (PLGA) with a lactic acid to glycolic acid ratio of 50:50, an acid end cap, and an average molecular weight (*M*_*w*_) of 100,000 Da was purchased from PolySciTech^®^ (West Lafayette, IN, USA). Tenofovir (TFV) was provided by CONRAD (Arlington, VA, USA). Dimethyl sulfoxide (DMSO) and hexafluoroisopropanol (HFIP) were purchased from VWR^®^ (Radnor, PA, USA) and Oakwood Laboratories (Wayne County, MI, USA). Phosphate-buffered saline (PBS) was purchased from Mediatech Inc. (Manassas, VA, USA). HPLC grade acetonitrile, trifluoroacetic acid, and HPLC grade water were purchased from Fisher Scientific (Pittsburgh, PA, USA). All materials were used as received without any further modification.

### 2.2 Preparation of electrospun PCL/PLGA fibers

To prepare polymer solutions for electrospinning, PCL and PLGA were dissolved in HFIP at 15% (w/v). PCL/PLGA blends, by adjusting the weight ratio of PCL to PLGA at 100:0, 80:20, 60:40, 40:60, 20:80, and 0:100, were dissolved in HFIP at 15% (w/v). All polymer solutions in their glass vials were placed on a ThermoScientific Labquake^TM^ tube rotator (Waltham, MA, USA) for overnight mixing to ensure complete dissolution of the polymer. TFV was added to polymer solutions at 10–40% (w/w)=wt% prior to electrospinning. All solutions were extruded from a 1 mL glass syringe, through a 21 G blunt-end needle at 20 μL/min, using a NE-1000 programmable single syringe pump (Farmingdale, NY, USA). During electrospinning, the applied voltage was 10 kV and the distance from the tip of the needle to the collector was maintained at 15 cm. Fibers were collected on a grounded stationary collector and/or a rotational drum covered with a wax paper. The rotational speed was 3000 rpm, and the corresponding linear rotational speed was 200 cm s^−1^ at the surface of the collector. Resulting fiber mats were dried and stored in a vacuum desiccator until used for analysis.

### 2.3 Mechanical testing

Mechanical tests were conducted on a single column screw-driven Instron^®^ 5943 universal materials testing machine (Norwood, MA, USA) under 24±1 °C and 45±5% RH in accordance with ASTM standard D5034-95. Dog-bone tensile specimens (ASTM standard D1708-96, 22 mm in nominal length and 5 mm in width) were carefully prepared by punching the electrospun fiber mats from a stainless steel die (ODC Tooling and Molds, Waterloo ON, Canada). Specimens with any visible defects were discarded, and a total of 5 specimens were used for each test group. Tensile tests were performed at a strain rate of 0.01 s^−1^ using Instron pneumatic clamps where load and displacement data were obtained. Young׳s modulus (linear region before 2% strain) and tensile strength (zero slope) were calculated from each corresponding stress–strain curve where the thickness of each sample was measured by a thickness gauge (MAPRA technik, Loughton, Essex, UK). Average thickness of the test specimens ranged from 0.05 mm to 0.15 mm. Data were analyzed using 2-way ANOVA (Prism, GraphPad).

### 2.4 Mechanical model

A mechanical model based on the results from average Young׳s moduli was used to validate drug partition in PCL/PLGA fibers. Average Young׳s moduli of blank PCL/PLGA fibers were fitted into rule of mixtures assuming the blend fiber system behaved similarly as a composite material. A Voigt model was used to simplify the interpretation of the results:

$E_{\mathrm{PCL}/\mathrm{PLGA}}=\left[E_{\mathrm{PCL}}\times \left(x\right)\right]+\left[E_{\mathrm{PLGA}}\times \left(1-x\right)\right]$, where x is the percentage concentration of PCL in blend fibers. Assuming drug–polymer interactions (e.g., TFV-PCL and TFV-PLGA) attributed to individual increases or decreases in Young׳s moduli of the blend fibers, the model was then modified as:

$$E_{\mathrm{overall}}=E_{\mathrm{PCL}/\mathrm{PLGA}}+\left({∆E}_{\mathrm{PCL}}\times \eta_{\mathrm{PCL}/\mathrm{TFV}}\right)+\left({∆E}_{\mathrm{PLGA}}\times \eta_{\mathrm{PLGA}/\mathrm{TFV}}\right),$$

where $\eta_{\mathrm{PCL}/\mathrm{TFV}}$ is the percentage of overall TFV partitioned in PCL and $\eta_{\mathrm{PLGA}/\mathrm{TFV}}$ is the percentage of overall TFV partitioned in PLGA.

### 2.5 In vitro drug release studies

1/4 in. diameter discs were taken from fiber mats using a metal die. Sample thickness was measured using a thickness gauge and initial sample mass was measured using a Mettler-Toledo XS105 analytical balance (Columbus, OH, USA). Fibers were placed into glass vials containing 10 mL of pre-warmed (37 °C) PBS release media (pH~7.2–7.4). Samples were kept at 37 °C in a rotary incubator at 200 rpm. At predetermined time points out to 240 h, 200 μL of liquid samples were placed into HPLC vials and replaced with fresh media to maintain sink conditions. HPLC samples were stored at 4 °C prior to analysis. The extent of drug release was expressed as percentage cumulative release and calculated as the concentration of drug in the release media relative to the initial drug concentration in the fibers.

HPLC analysis was used to quantify tenofovir concentration in the release media. A Shimadzu Prominence LC20AD UV-HPLC system equipped with a Phenomenex Kinetex C18 column (5 µm, 250×4.6 mm) and LCSolutions software were used to quantify drug levels in samples. The HPLC mobile phase consisted of a 72% HPLC graded H_2_O with 0.045% trifluoroacetic acid buffer and 28% acetonitrile with 0.036% trifluoroacetic acid buffer. Tenovofir was freely soluble in the mobile phase. The HPLC methods included 30 °C column temperature, 1 mL/min flow rate, 10 min run time, 20 μL sample injection volume and UV/vis detection at 259 nm. Tenofovir standard curves were prepared by dissolving 25 mg of tenofovir in PBS to a concentration of 200 μg/mL and diluting serially at 1:2 until concentrations of 0.1 ug/mL. Later, the tenofovir standards and unknown samples were detected by UV-HPLC as described previously (Carson et al., 2016). Drug loading (encapsulation efficiency) was quantified by comparing drug-containing fibers dissolved in DMSO to a tenofovir standard curve in DMSO (0.1–500 μg/mL). Results were the average of three independent measurements.

### 2.6 Materials characterization

Surface morphologies of electrospun nanofibers were investigated using a scanning electron microscopy (SEM). Circular punches were taken from the fiber mats and sputter coated with Au/Pd for 90 s using a SPI sputter-coater (West Chester, PA, USA) at 80–100 mTorr. SEM micrographs were acquired from a FEI Sirion SEM system (UW, NanoTech User Facility) at 5 kV, using a spot size 3, and a working distance of 5.0 cm. Post-stretched fiber images were taken at near or less than 1 mm from the fracture site (*n*=100). Image analysis was carried out by image processing software (ImageJ, National Institutes of Health, Bethesda, MD, USA) using OrientationJ plugin for fiber alignment measurement by applying a Riesz filter at 70% minimal coherency and 2% minimal energy.

To measure sample shrinkage and mass loss after removing them from release media, dogbone samples were allowed to air dry in laboratory atmosphere. Square portions (clamping area) of the dogbone samples were used for sample shrinkage measurement with the assumption that shrinkage of the sample was proportional within the samples. Mass of the sample was weighted daily and mass values were accepted with less than 1% change after 24 h. Percentage shrinkage and mass loss were calculated based on the following equation:

$$\mathrm{Shrinkage}\;\left(\%\right)=\frac{A_{D}-A_{i}}{A_{i}}\times 100$$

$$\mathrm{Mass}\;\mathrm{loss}\;\left(\%\right)=\frac{W_{D}-W_{i}}{W_{i}}\times 100,$$

where *A*_*D*_ is the dried square clamping area of the dogbone samples, *A*_*i*_ is the initial area, *W*_*D*_ is the dried weight, and *W*_*i*_ is the initial weight. For mechanical testing on dogbone specimens after removing from the release media, new nominal length and width after shrinkage was used for mechanical property analysis. Each sample was set for its corresponding nominal length for accurate strain comparison.

### 2.7 Statistical analyses

Unless specified, data were described as mean±standard deviation. One-way analyses of variance (ANOVA) were performed for comparative studies at an acceptable significance level of *P*<0.05.

## 3 Results

### 3.1 Stress–strain curves of PCL/PLGA fibers

**Fig. 1:**
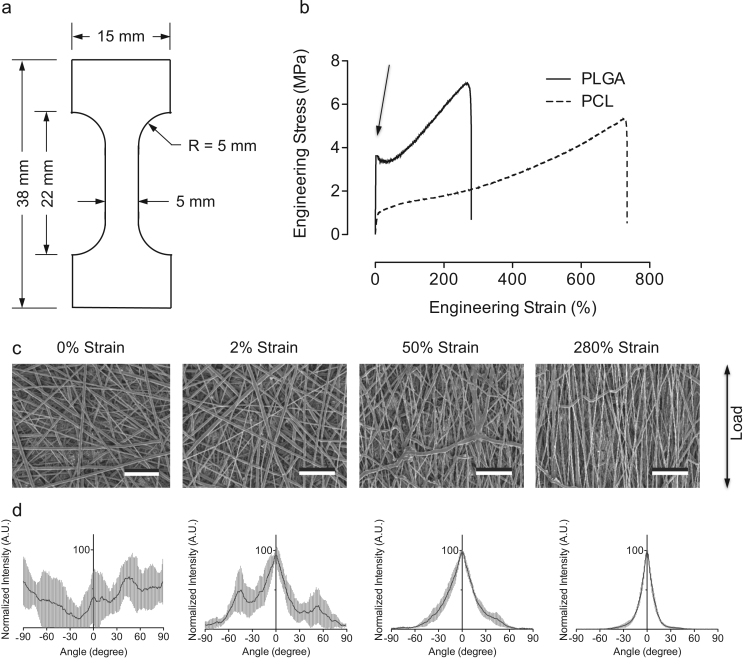
(a) Schematics of a dogbone specimen and its dimension used in this work. (b) Typical stress strain curves of electrospun randomly oriented PCL (dashed line) and PLGA (solid line) fibers, where PLGA fibers exhibit a necking behavior (arrow pointing). (c) SEM images of PLGA fiber alignment due to mechanical stretching at 0%, 2%, 50%, and 280% strain. An arrow indicates load-applying direction. Scale bar=20 μm. (d) Distribution plots of fiber alignment from the acquired SEM images. Load-induced fiber alignment was analyzed by ImageJ software with OrientationJ plugin. Results suggested increasing strain increased fiber alignment. Gray area represents standard deviation (*n*=5).

Fundamental studies on mechanical properties of electrospun nanofibers typically focus on uniaxial tensile testing of randomly orientated or aligned fiber configurations. In this study, blends of PCL/PLGA nanofibers were electrospun at various PCL to PLGA ratios followed by mechanical stretching of the as-punched dogbone specimens under a uniaxial load in tension (Fig. 1a). Representative stress–strain curves of randomly orientated PCL and PLGA electrospun nanofibers highlight features that are intrinsic to the mechanical behaviors of these polyesters (Fig. 1b). Using PLGA nanofibers as an example, the stress–strain curve can be divided into an elastic, yielding and strain-hardening region. Specifically, the initial linear elastic region occurs up to 2% strain where stretching of the fiber mats results in slight alignment of the fiber network to the applied load direction (Fig. 1c). Increasing applied strain to 50% and 280% significantly increased fiber alignment to 30° and 14° in full width at half-maximum, respectively (Fig. 1d). Additionally, a noticeable necking phenomenon is observed from PLGA samples indicating the onset of plastic deformation in mechanical behavior. In general, PLGA samples are stiffer and stronger than PCL samples. By contrast, PCL appears to be more ductile than PLGA.

### 3.2 Mechanical properties of PCL/PLGA/TFV fibers

**Fig. 2:**
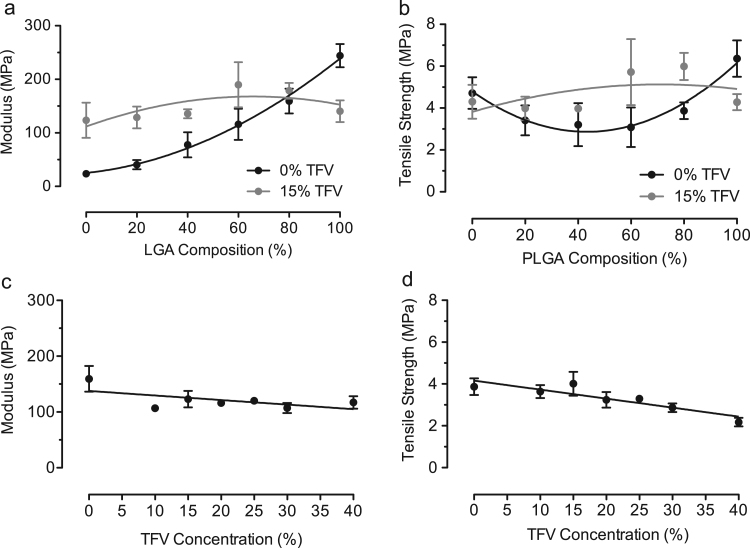
Mechanical properties of blank PCL/PLGA fibers (black dots) and fibers loaded with 15 wt% of TFV (gray dots): (a) Young׳s modulus and (b) tensile strength. Mechanical properties of 20PCL/80PLGA fibers loaded with TFV up to 40 wt%: (c) Young׳s modulus and (d) tensile strength.

We measured the Young׳s modulus and tensile strength of PCL/PLGA blend fibers alone and after incorporating TFV to understand the effect of drug loading on fiber mechanical properties. Yield strength, elongation to failure, and toughness (work to failure) of PCL/PLGA/TFV fibers are reported in Supplementary Information. The average Young׳s moduli of PCL and PLGA nanofibers were 24±4 MPa and 244±22 MPa, respectively (Fig. 2a). For the blended polymer fibers, increasing PLGA composition from 0% to 100% significantly increased the average Young׳s modulus by approximately five-fold due to the high inherent stiffness of PLGA (*P*<0.05). By contrast, we did not observe the same magnitude increase in the average Young׳s moduli as a function of PLGA content once the polymer blend fibers were loaded with 15 wt% of TFV. Our results suggests that the stiffening mechanism due to increasing PLGA content in PCL/PLGA fibers was offset by the incorporation of TFV at a level of 15 wt%, which alone significantly reduced the ductility of the fibers (see Supplementary Figure S1). Unlike Young׳s modulus, average tensile strength of PCL/PLGA fibers at various PLGA compositions showed minimal effects on fiber composition or incorporation of TFV at 15 wt% (Fig. 2b). Overall, average tensile strength of PCL/PLGA/TFV fibers ranged from 3.1±0.9 MPa to 6.4±0.9 MPa.

We demonstrated previously that PCL/PLGA (20/80) fibers provide zero-order drug release when loaded with 15 wt% TFV (Carson et al., 2016), and selected this composition to measure the effect of various TFV drug loading on mechanical properties of the bulk mesh. Within this polyester composition, we observed that the average Young׳s modulus (Fig. 2c) and tensile strength (Fig. 2d) remained constant at all TFV loadings from 0 wt% to 40 wt%. Although the Young׳s modulus and average tensile strength appeared to decrease slightly with increasing TFV loading, the correlation was insignificant. Overall, our results showed that the modulus and tensile strength of PCL/PLGA (20/80) fibers were independent of TFV loading up to 40 wt%.

### 3.3 Effect of aligned and random PCL/PLGA fibers

**Fig. 3:**
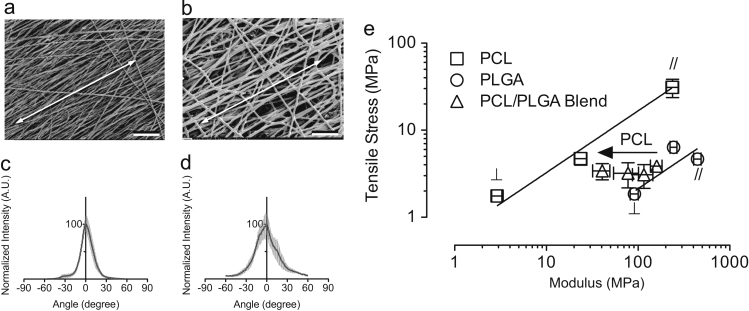
SEM image of aligned fibers (a) PCL and (b) PLGA collected from a rotation drum. An arrow indicates the drum rotating direction. Scale bar=100 μm. Distribution plots of (c) PCL and (d) PLGA fiber alignment from the acquired SEM images. (e) Correlation of tensile stress – Young׳s modulus for aligned blank PCL/PLGA fibers showing effects of polymer composition and fiber alignment (collected from a rotation drum) on their mechanical properties. Within the upper and lower boundaries, fiber mechanical properties are tunable through fiber composition and fiber alignment.

Structure-property relationships of random and aligned fibers have been reported previously (Katta et al., 2004, Li et al., 2007, Stylianopoulos et al., 2008). Here, we measured Young׳s modulus and tensile strength of aligned PCL and PLGA fibers and compared their values to the same properties obtained from various PCL/PLGA blend fibers. In addition, separate sets of PCL and PLGA fibers were collected on a rotation drum and mechanically tested in parallel and in perpendicular to the fiber alignment direction (drum rotating direction) (Figs. 3a and b). The degree of fiber alignment appeared to depend on productivity of the fibers during electrospinning, with higher productivity inversely correlated with higher alignment (Fig. 3c and d). Both PCL and PLGA fibers showed the highest tensile strength when fiber orientation was pre-aligned parallel to the applied load direction (Fig. 3c). By contrast, the lowest tensile strength was obtained from fibers orientated perpendicular to the applied load direction. Furthermore, the tensile strength of randomly oriented fibers fell in between the values of pre-aligned fibers. As noted previously, we observed that varying PCL and PLGA composition in the blend fibers significantly affected fiber stiffness whereas tensile strength remained unchanged. In addition to the composition effect, fiber alignment also had a significant impact on the stiffness of the samples. Trend lines between PCL and PLGA fibers showed the lower and upper boundaries of tensile strength and Young׳s modulus for this particular blend fiber system. Therefore, within these boundary limits, fibers of particular strength and stiffness can be tailored based on the degree of alignment and the composition of PCL and PLGA.

### 3.4 Fiber structure and morphology

**Fig. 4:**
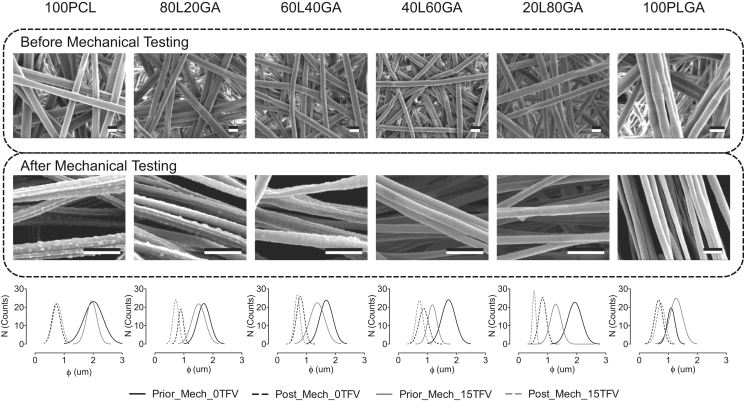
SEM images of PCL/PLGA fibers loaded with 15 wt% of TFV before and after tensile testing (L=PCL; GA=PLGA). Scale bar=2 μm. Aggregates on the surface of 100PCL and 80L20GA fibers after mechanical stretching to failure are noticeable. The amount of the surface aggregates decreases as increasing PLGA composition in the fiber. Fiber diameter decreased as a result of mechanical stretching (*n*=100).

Changes in PCL/PLGA/TFV fiber morphology were investigated using SEM images after mechanical testing to investigate drug polymer compatibility (Fig. 4). Blend fibers without TFV exhibited a smooth and defect-free surface with no morphological changes after mechanical testing (data not shown). Conversely, after mechanical stretching, we observed that PCL fibers (100% and 80% content) loaded with 15 wt% of TFV displayed surface aggregates (*D*=135±35 nm). The amount of surface inhomogeneities appeared to decrease with increasing PLGA content in the fibers. This observation may suggest that TFV is more compatible with PLGA than PCL due to the excess of carbonyl groups in PLGA that can serve as hydrogen bonding sites for TFV. Fiber diameters were 2.0±0.3 μm and 1.1±0.1 μm for PCL and PLGA fibers respectively whereas fiber diameter decreased to 0.7±0.2 μm after mechanical stretching. It seems that micro-defects (e.g., cracks) formed around this fiber diameter, which would limit the ability to further reduce fiber diameter by continued stretching. In addition, elongation of PLGA fibers is expected to be less than that of PCL fibers (see Supplementary Figure S1) since PLGA originally has a smaller fiber diameter than PCL fibers. The reduction of the fiber diameter is related to the total allowable amount of the strain that can be applied to the fibers.

### 3.5 Mechanical properties after in-vitro release

**Fig. 5:**
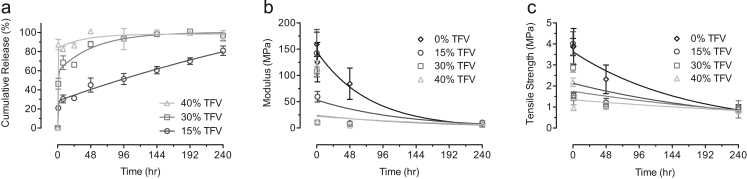
Decoupling losses of mechanical properties due to effects of TFV release and polymer degradation. (a) TFV cumulative release curves for 20PCL/80PLGA fibers loaded with 15, 30, and 40 wt% of TFV. Release profiles switched from a sustained release (15 wt% TFV) to a burst release at higher drug loading (30 and 40 wt% TFV). (b) Average Young׳s modulus of 20PCL/80PLGA fibers with and without TFV loading after in the release media for 1, 48, and 240 h. The loss of Young׳s modulus at early time point is associated with release of TFV. At prolonged time point, drug-load fibers exhibit a similar Young׳s modulus as the blank fibers. (c) Average tensile strength of 20PCL/80PLGA fibers with and without TFV loading after in the release media for 1, 48, and 240 h. A similar trend of losses in tensile strength can be seen for the blank and drug-loaded fibers.

We have previously shown that PCL/PLGA (20/80) fiber formulations sustain TFV release over 10 days in vitro (70% cumulative release), and we show here minimal changes in mechanical properties of this composition even with high TFV loading. We observed that increasing TFV concentrations from 15 wt% to 40 wt% in the fibers increased the burst release magnitude of TFV from 20% to 85%, respectively (Fig. 5a). These values are higher than our previous reported data perhaps due to differences in sample shape and thickness (Carson et al., 2016). We also investigated the effect of drug release on mechanical properties by measuring Young׳s modulus and tensile strength at early time points prior to any significant polymer degradation (*t*_1/2_=30 days for PLGA and *t*_1/2_>18 months for PCL) (Lu et al., 1999, Park, 1995, Peña et al., 2006). Uniaxial tensile tests were performed after 1, 48, and 240 h in the release media for both blank and TFV-released PCL/PLGA (20/80) samples. We observed that the average Young׳s modulus and average tensile strength of the blank PCL/PLGA (20/80) fibers decreased significantly in PBS over time (Fig. 5b and c). Average Young׳s moduli decreased by 20% after 1 h followed by an additional 30% after 48 h and 90% after 240 h in the release media (*P*<0.05). In addition, average tensile strength of the same fibers remained at a similar value after 1 h followed by a significant decrease of 40% after 48 h and an additional 40% after 240 h (*P*<0.05). For drug-loaded fibers, we observed significant decreases after 1 h in the average Young׳s moduli of 60%, 90%, and 90% for 15, 30, and 40 wt% TFV loading, respectively (*P*<0.05). In addition, average tensile strength of the same fibers showed significant decreases of 60%, 50%, and 50% after 1 h (*P*<0.05). At the end of the study (e.g., 240 h), the average Young׳s modulus and tensile strength of the drug-incorporated fibers reduced 90% and 80%, respectively. In general, we show that a significant decrease in mechanical properties is associated with drug release from electrospun fibers.

### 3.6 Fiber structure after in-vitro release

**Fig. 6:**
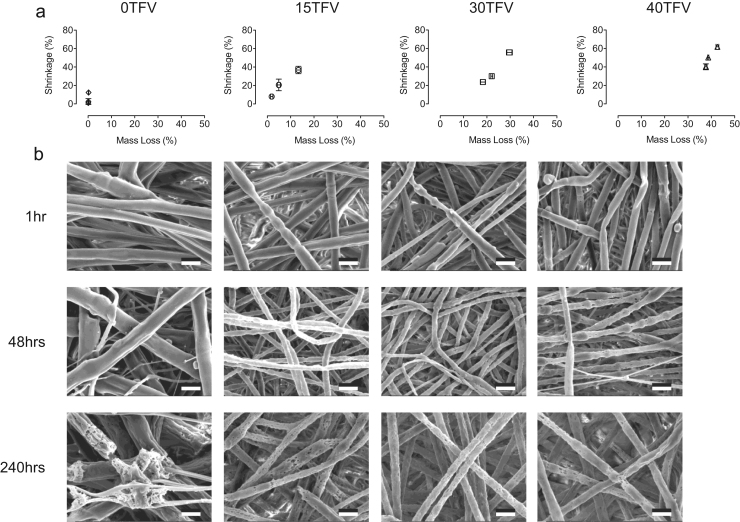
(a) Percentage shrinkage and mass loss of 20PCL/80PLGA fibers with and without TFV after 1, 48, and 240 h in the release media. A linear correlation of sample shrinkage and mass loss is observed. (b) SEM images of 20PCL/80PLGA fibers with and without TFV loading after in the release media for 1, 48, and 240 h followed by mechanical testing. Scale bar=2 μm.

Given that changes in physical characteristics affect mechanical performance of the drug-eluting fibers, we measured sample shrinkage, mass loss, and fiber structure to correlate with loss of mechanical properties during drug release. We observed that shrinkages of PCL/PLGA (20/80) fiber samples after 240 h were 37±4%, 56±1%, and 62±3% when loaded with 15, 30, and 40 wt% of TFV, respectively (Fig. 6a). In addition, fibers in the release media after 240 h experienced mass losses of 13±1%, 30±1%, and 43±1% when loaded with TFV at 15, 30, and 40 wt%, respectively. According to the in vitro release data (Fig. 5a), fibers loaded with 15, 30, and 40 wt% achieved a TFV cumulative release of 80±5%, 97±5%, and 96±6% at 240 h, respectively. Our data on the mass loss support the in vitro release results, suggesting that the release of TFV attributes to the majority of mass loss in the drug-eluting fibers. Furthermore, fiber morphology examined by SEM showed surface characteristic changes including nodules, pores, and hollow sites (Fig. 6b). Of particular importance, these surface defects result in stress concentration during mechanical stretching and consequently becoming potential weak spots for failure to occur. This explains the significant loss of mechanical properties of the blank PCL/PLGA (20/80) fibers after 240 h in the release media (Fig. 5b and c). In general, sample shrinkage, mass loss, and surface defects of the PCL/PLGA (20/80) fibers with and without TFV are observed and contribute to the overall decrease in measured mechanical properties.

### 3.7 In vitro TFV release after mechanical stretching of the fibers

**Fig. 7:**
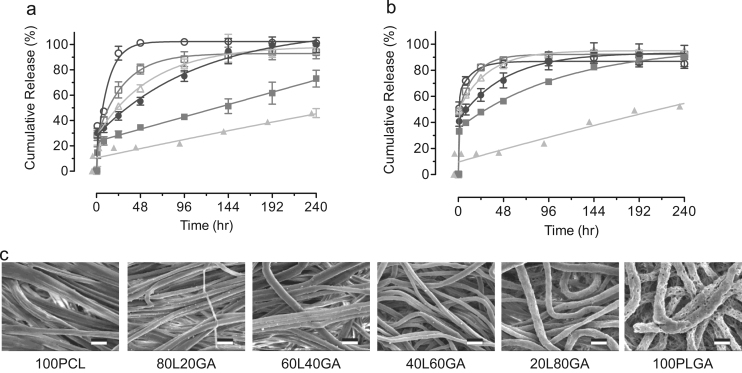
Cumulative release curves of PCL/PLGA blend fibers loaded with 15% TFV (a) prior to mechanical stretching (b) after mechanical stretching to failure. (*Open circles*) 100PCL, (*Open squares*) 80PCL/20PLGA, (*Open triangles*) 60PCL/40PLGA, (*Filled circles*) 40PCL/60PLGA, (*Filled squares*) 20PCL/80PLGA, and (*Filled triangles*) 100PLGA. (c) SEM images of PCL/PLGA blend fibers mechanically stretched followed by a 10-day in vitro release study. Note that 100PCL fibers showed surface aggregates after mechanical stretching (Fig. 4) whereas the aggregates were not seen here after the release study. Scale bar=2 μm.

We performed in vitro drug release studies on TFV-loaded (15 wt%) PCL/PLGA samples at various compositions after tensile tests to show changes in release profiles due to mechanical deformation. Sustained release of TFV depended on PLGA content in PCL/PLGA fibers before mechanical stretching (Fig. 7a). By contrast, fibers being stretched to failure showed increases in release rates where PCL/PLGA (20/80) fibers reached 70% cumulative release at 96 h as compared to the same level of cumulative percentage release at 240 h without mechanical stretching (Fig. 7b). Changes in TFV release profiles are perhaps due to decreased fiber diameters (Fig. 4) resulting in an increase in the surface area of the fabrics. Furthermore, stretched fibers have a smaller fiber diameter that decreases the distance for core-partitioned TFV to diffuse. In addition, stretched fibers in the release media at 240 h were free of surface aggregates as observed from SEM images (Fig. 7c), suggesting that the surface aggregates (Fig. 4) may be attributed to TFV. In addition, in vitro drug release studies indicated an above 90% cumulative release of TFV for PCL and PCL-rich fibers at 240 h (Fig. 7b), which resulted in the disappearance of TFV surface aggregates. At high PLGA concentrations, the surface morphologies and fiber structure appeared to be similar to our previous observations (Fig. 6). Surface defects due to TFV release can also be seen on the PLGA and PLGA-rich fibers. In general, mechanical stretched fibers exhibit a faster release rate.

### 3.8 TFV partitioning probed by mechanical testing

**Fig. 8:**
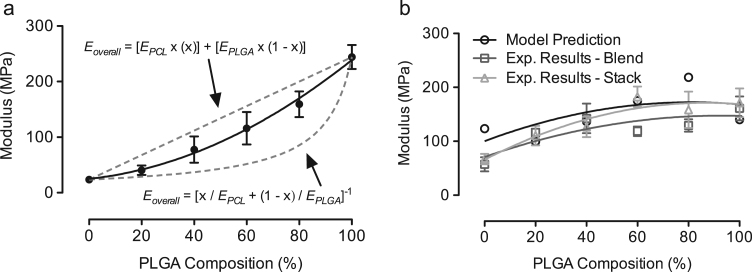
Prediction of TFV partitioning using a mechanical model. (a) Rule of mixtures showing PCL/PLGA blend fibers fell in between the upper bound limit (Voigt model: axial loading) and the lower bound limit (Reuss model: transverse loading). (b) Correlations between model prediction of TFV partitioning: 80% of TFV is in PCL and 20% of TFV is in PLGA. Experimental results on blend fiber loaded with TFV, and experimental results on stacked fiber pre-loaded with the exact amount of TFV in either PCL or PLGA fibers.

We developed a mechanical model to quantify TFV partitioning in the electrospun PCL/PLGA fibers to understand the correlations between mechanical properties, TFV release rates, and drug and polymers interactions. The average Young׳s moduli of blank PCL/PLGA fibers were within the upper and lower bound limits from rule of mixtures (Fig. 8a). In addition, the model predicts 80% of the total TFV is associated with PCL and 20% of the total TFV is associated with PLGA, which is in good agreement with the experimental results from the blend PCL/PLGA/TFV fibers (Fig. 8b). To verify these results, we individually electrospun PCL/TFV and PLGA/TFV fibers with predetermined amounts of TFV as predicted by the model and stacked them into a single composite mat. Results from mechanical testing of the stacked fibers (Fig. 8b), consisting 80% of the TFV in PCL fibers and 20% of the TFV in PLGA fibers, showed good agreement with the model prediction. In addition, average Young׳s moduli of the stack fibers exhibited comparable values as the experimental results from PCL/PLGA blend fibers loaded with 15 wt% of TFV. In general, we report a mechanical model to estimate TFV partitioning in PCL/PLGA electrospun blend fibers.

## 4 Discussion

In this work, we correlate mechanical properties of PCL/PLGA blend fibers with TFV release. We showed that increasing PLGA composition offsets the stiffening attributed to incorporation of TFV in the blend fibers. Furthermore, drug release significantly decreased the mechanical properties of PCL/PLGA (20/80) fibers. Moreover, a mechanical model was used to infer preferential partitioning of TFV into the PCL phase compared to the PLGA phase of blended fibers, which we further confirmed experimentally with stacked composite fibers. Our findings provide insights to drug–polymer interaction, drug loading, and drug partitioning using electrospun blend polymer fibers. In addition, our results may inform the design of tissue engineered biomaterial scaffolds that have dual requirements for maintaining mechanical properties while also delivering agents (e.g., growth factors) that upon release leads to substrate degradation overtime.

Mechanical properties of PCL/PLGA fibers suggested that PCL fibers had a lower Young׳s modulus than PLGA fibers (10-fold) while increasing PLGA content increased average Young׳s moduli of the blend fibers. Several studies report on the dependence of fiber diameter on the average Young׳s modulus of PCL fibers (Croisier et al., 2012, Jordan and Korley, 2015, Tammaro et al., 2015). In addition, tensile strength of the PCL fibers appeared to be independent of fiber diameter. By contrast, PLGA exhibits a wide range of mechanical properties mainly due to its lactic to glycolic acid ratios (Li et al., 2006, Li et al., 2002, Meng et al., 2010). The observed higher Young׳s modulus of PLGA as compared to PCL is in agreement with literature. In addition, mechanical properties of PCL/PLGA blend fibers exhibited a gradual increase in Young׳s modulus with increasing PLGA content in the fibers (Fig. 2a), which has also been observed by others (Bianco et al., 2013; Liang et al., 2013; Torricelli et al., 2014). In a previous study, the tensile strength of PCL/PLGA (80/20) fibers were reported to be 2–3 MPa (Franco et al., 2011). Our results are in agreement with these reported values. In general, PLGA has a higher intrinsic mechanical stiffness and strength than PCL. Increasing PLGA composition in the blend fibers results in a gradual increase in mechanical properties, suggesting that the two polymers are miscible.

PCL/PLGA fibers incorporating TFV at various loadings were investigated to show effects of drug–polymer interactions on mechanical properties. Changes in average Young׳s moduli of drug-loaded fibers indicated a high amount of drug–polymer interactions (Fig. 2a). A previous study using PLA/PEG (87/13) fibers loaded with 10 wt% of 5-nitro-8-hydroxyquinoline, an antibacterial drug, showed significant decreases in Young׳s modulus due to drug–polymer interactions (Toncheva et al., 2016). Others have also reported changes in Young׳s modulus for PLGA fibers loaded with small molecule drugs (Mo et al., 2015, Ranjbar-Mohammadi et al., 2016). By contrast, an increase in elastic modulus was reported for PCL fiber loaded with 5 wt% of linezolid (Tammaro et al., 2015). Collectively, these findings support that drug–polymer interactions affect mechanical properties of electrospun polyester fibers. In addition, drug–polymer interactions become more pronounced at higher loading as expected and was suggested in a study using metronidazole loaded up to 40 wt% in PCL/gelatin (50/50) fibers (Xue et al., 2014). Our results showed that increasing TFV loading (up to 40 wt%) has minimal effects on elastic modulus and tensile strength of the PCL/PLGA (20/80) fibers due to offsetting drug–polymer interactions (Fig. 2c). In general, drug–polymer interactions significantly affect mechanical properties of electrospun blend fibers while increasing drug loading may magnify these interactions and reduce the corresponding mechanical properties.

We hypothesized that the mechanical performance of polymer fibers significantly decreases due to their degradation in simulated physiological conditions. For example, polyester fibers such as poly(lactic acid) (PLA) and poly(glycolic acid) (PGA) resorbed and degraded in vivo resulting in low mechanical properties (Majola et al., 1991, Paganetto et al., 1991). In a previous study, biodegradable polyester-urethane fiber scaffolds consisting of poly[3-(*R*-hydroxybutyrate)-*co*-(ε-caprolactone)]-diol and poly[ε-caprolactone-*co*-glycolide]-diol linked with 2,2,4-trimethylhexamethylene diisocyanate were mechanically stretched in uniaxial tension after a predetermined time of degradation in PBS (Limbert et al., 2016). The average Young׳s modulus and tensile strength decreased 20% and 50% after 4 days of degradation and an additional 20% and 40% by 24 days, respectively. Changes in mechanical properties during degradation have been attributed to hydrolysis of the segmented structure of PU-based materials. Furthermore, cleavage of the ester linkages resulting in fragmentation of the fibers provides direct evidence of degradation. Another possible degradation mechanism includes erosion of the fiber surfaces. For example, PLA/PCL (90/10) fibers showed a 10% loss in mass leading to an 80% decrease in strength over 16 weeks of investigation in PBS (Vieira et al., 2011). Our results for blank PCL/PLGA (20/80) fibers showed a 10% mass loss with a decrease of 80% in strength after 10 days in PBS. Although the loss of strength in our study is comparable with previous results, our data suggest a faster degradation rate. The differences are perhaps due to a higher hydrolytic damage on PLGA than PLA. Overall, polymer degradation plays an important role in the decrease of mechanical properties.

Fiber mechanical properties are also significantly affected by drug release. Our results suggests that TFV release resulted in significant loss of mechanical properties at early time points (e.g., 1 h and 48 h). By contrast, at later time points (240 h) when cumulative TFV release reached above 80%, loss of mechanical properties in drug-loaded fibers were comparable to the blank fibers. These results suggest that fiber degradation dominated the mechanical attributes at these later times. Furthermore, the decrease in mechanical properties appeared to be faster in fibers with a higher drug loading (e.g., 30 wt% and 40 wt%), indicating a loss of mechanical property related to release. We also measured TFV release from PCL/PLGA fibers after being mechanically stretched. Our results show that stretched fibers release drug faster than the non-stretched fibers. From a design perspective for drug-eluting fibers, changes in release rate due to mechanical disturbance may promote an undesired daily dosage. Therefore, future work may be necessary to decouple the dependence between drug release and mechanical properties.

In blend polymer fibers, drug partitioning was often observed in conjunction with tunable release rates, such as in the case of poly(ε-caprolactone) and poly(oxyethylene- b-oxypropylene-b-oxyethylene) blend fibers (Natu et al., 2010). Similarly, burst of lysozyme was suggested from partitioning in water soluble poly(ethylene oxide) (PEO) blended with polyester-based polymers fibers (Kim et al., 2007). However, quantification of drug partitioning is deemed to be very difficult using release kinetics alone since many factors (e.g., surface drug, wetting, erosion, diffusion, etc.) are interrelated. In this study, we established a mechanical model and used it to predict drug partitioning in blended PCL/PLGA 80/20 fibers. Our mechanical model was based on the elastic modulus in composite theory with additional terms (i.e., individual increase or decrease in modulus after drug incorporation) for the Voigt model. We validated TFV partitioning predicted from our model by using pure PCL or PLGA fibers individually loaded with predetermined drug content and stacked them into a composite. The mechanical properties of the stacked materials were similar to the blend fibers. To our knowledge, this is the first report quantifying drug partition in blend electrospun nanofibers using mechanical testing. Our model provides an easy and straightforward method to predict drug partitioning in electrospun polymer blend fibers. Overall, drug partitioning has significant impact on release profiles and mechanical properties of electrospun blend polyester fibers. The current mechanical model may provide a rapid and direct method to examine drug partitioning.

## 5 Conclusions

In summary, we report correlations between mechanical properties and drug release rates of electrospun blend fibers. Our results showed that incorporating TFV into PCL/PLGA fibers significantly modified the mechanical properties from blank fibers, suggesting a high level of drug-polymer interactions. TFV release decreased mechanical properties significantly at early time points. Pre-stretched PCL/PLGA blend fibers to failure showed higher release rates as compared to the non-stretched samples. Drug partitioning in PCL/PLGA fibers was evaluated using a mechanical model, and experimental data using stack fiber configurations confirmed that 80% of the TFV is in the PCL phase and 20% of TFV is in the PLGA phase. Our study contributes to scientific understanding of mechanical performance of drug-eluting fibers.
