# Supplementary material for: Relationships between mechanical properties and drug release from electrospun fibers of PCL and PLGA blends
Source: J Mech Behav Biomed Mater. 2017 Jan;65:724–33. doi: 10.1016/j.jmbbm.2016.09.004 (PMC6461716; doi:10.1016/j.jmbbm.2016.09.004)
Supplement: Supplementary file 1 — Supplementary material [file mmc1.docx]

Supporting Information to the Manuscript

**Relationships between mechanical properties and drug release from electrospun fibers of PCL and PLGA blends**

Shih-Feng Chou^1^ and Kim A. Woodrow^1,†^

Author Affiliation:

^1^Department of Bioengineering, University of Washington

3720 15^th^ Ave NE, Seattle WA, 98195-5061, USA

^†^Corresponding author:

Dr. Kim A. Woodrow

Foege N410D, Department of Bioengineering, University of Washington

3720 15^th^ Ave NE, Seattle WA, 98195-5061, USA

1-(206)-685-6831

woodrow@uw.edu

**Tensile Properties**

**Figure S1.** Tensile properties of PCL/PLGA blend fibers at various PLGA concentrations: (a) yield strength, (b) fracture strain, and (c) toughness (work to fracture). Tensile properties of PCL/PLGA (20/80) fibers at various TFV loading: (d) yield strength, (e) fracture strain, and (f) toughness (work to fracture).

**Differential Scanning Calorimetry**

**Table I.** Percentage Change in TFV crystallinity before and after mechanical testing

| PCL/PLGA | 100/0 | 80/20 | 60/40 | 40/60 | 20/80 | 0/100 |
| --- | --- | --- | --- | --- | --- | --- |
| 230C | -0.1% | -0.1% | -0.2% | -0.3% | 0% | - |
| 290C | 0% | -0.4% | -0.2% | -0.2% | -0.5% | -0.4% |
| Combined | -0.1% | -0.5% | -0.5% | -0.6% | -0.5% | -0.4% |

**Table II.** Raw Data in Percentage Crystallinity of TFV.

|  | 230C | | 290C | | Combined | |
| --- | --- | --- | --- | --- | --- | --- |
| PCL/PLGA | Before | After | Before | After | Before | After |
| 100/0 | 0.26% | 0.17% | 0.28% | 0.24% | 0.55% | 0.40% |
| 80/20 | 0.28% | 0.15% | 0.41% | 0.02% | 0.68% | 0.17% |
| 60/40 | 0.39% | 0.16% | 0.25% | 0.02% | 0.64% | 0.18% |
| 40/60 | 0.56% | 0.21% | 0.26% | 0.02% | 0.82% | 0.23% |
| 20/80 | 0.16% | 0.16% | 0.60% | 0.07% | 0.74% | 0.23% |
| 0/100 | 0 | 0 | 0.79% | 0.41% | 0.77% | 0.40% |

**Figure S2.** Differential scanning calorimetry thermograms of blend PCL/PLGA fibers at various compositions loaded with TFV before and after mechanical testing.

**High Performance Liquid Chromatography**

|  | 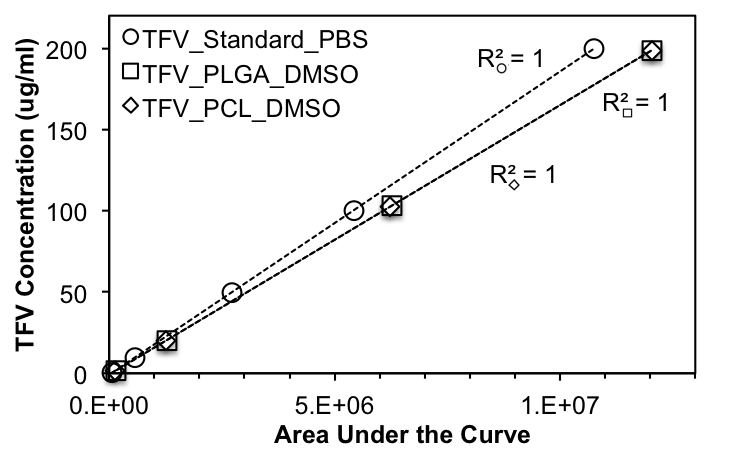 |
| --- | --- |
| (a) | (b) |

**Figure S3.** Representative HPLC calibration curves (a) TFV in PBS and (b) PCL/TFV and PLGA/TFV in DMSO as compared to TFV in PBS.
